# Supplementary material for: Pan-Cancer Drug Sensitivity Prediction from Gene Expression using Deep Learning
Source: bioRxiv. 2024 Nov 15:2024.11.15.623715. Preprint. [Version 1] doi: 10.1101/2024.11.15.623715 (PMC11601385; doi:10.1101/2024.11.15.623715)
Supplement: Supplement 1 — Document S1. Extended Data Fig. 1–3 and Tables S1–S11. [file media-1.pdf]

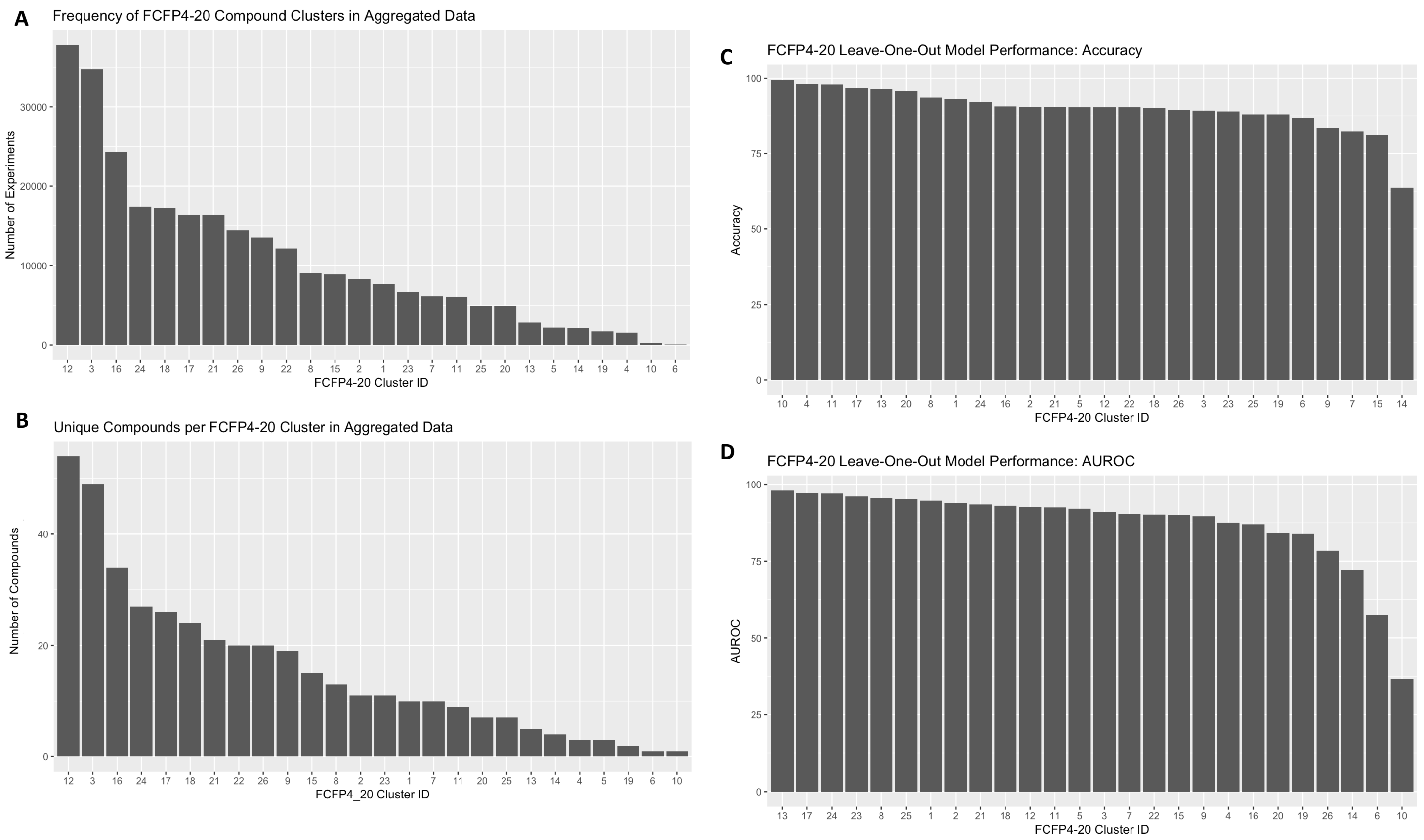

Extended Data Fig. 1. Small molecule compound groups clustered by FCFP4-20 fingerprints distribution and compound cross-domain validation model performance results. (A) Distribution of compound groups clustered by FCFP4-20 fingerprints in aggregated model development dataset. (B) Representation of FCFP4-20 fingerprint compound clusters in aggregated model development dataset. (C) Compound FCFP4-20 fingerprint cluster cross-validation leave-one out model performance accuracy by fingerprint cluster group. (D) Compound FCFP4-20 fingerprint cluster cross-validation leave-one out model performance AUROC by fingerprint cluster group.

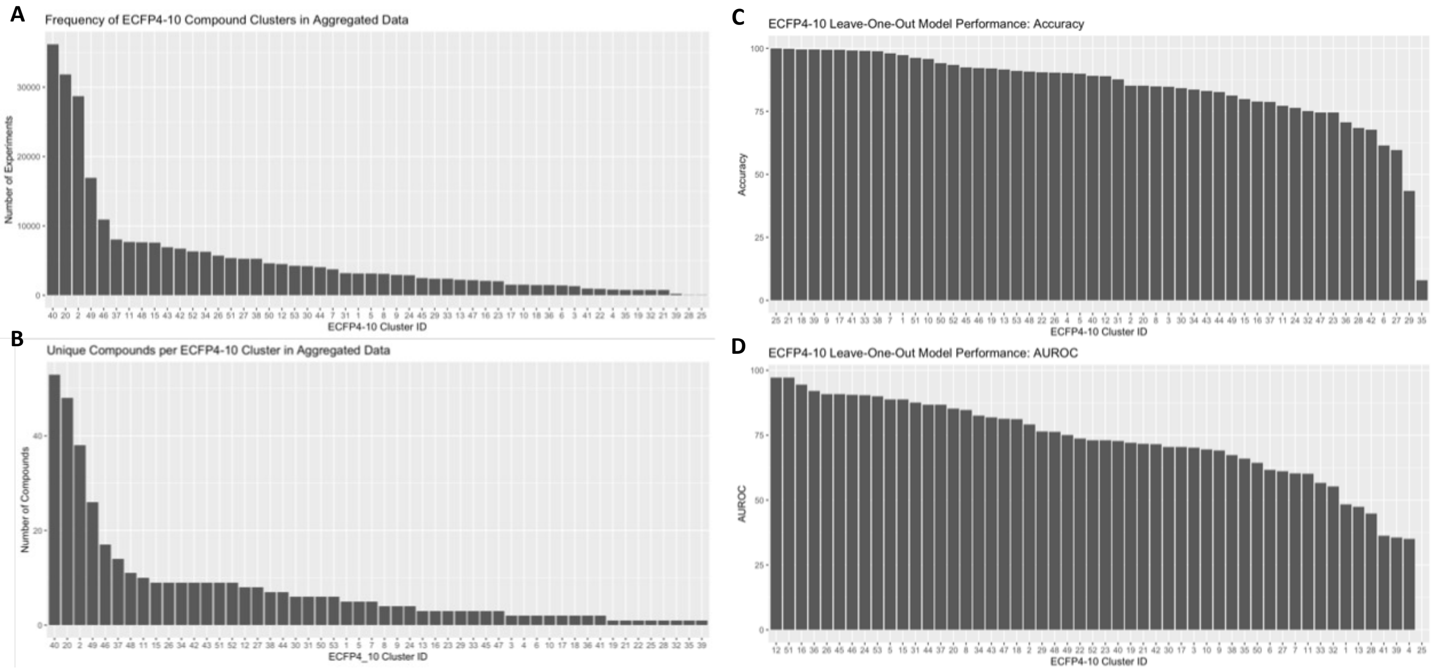

Extended Data Fig. 2. Small molecule compound groups clustered by ECFP4-10 fingerprints distribution and compound cross-domain validation model performance results. (A) Distribution of compound groups clustered by ECFP4-10 fingerprints in aggregated model development dataset. (B) Representation of ECFP4-10 fingerprint compound clusters in aggregated model development dataset. (C) Compound ECFP4-10 fingerprint cluster cross-validation leave-one out model performance accuracy by fingerprint cluster group. (D) Compound ECFP4-10 fingerprint cluster cross-validation leave-one out model performance AUROC by fingerprint cluster group.

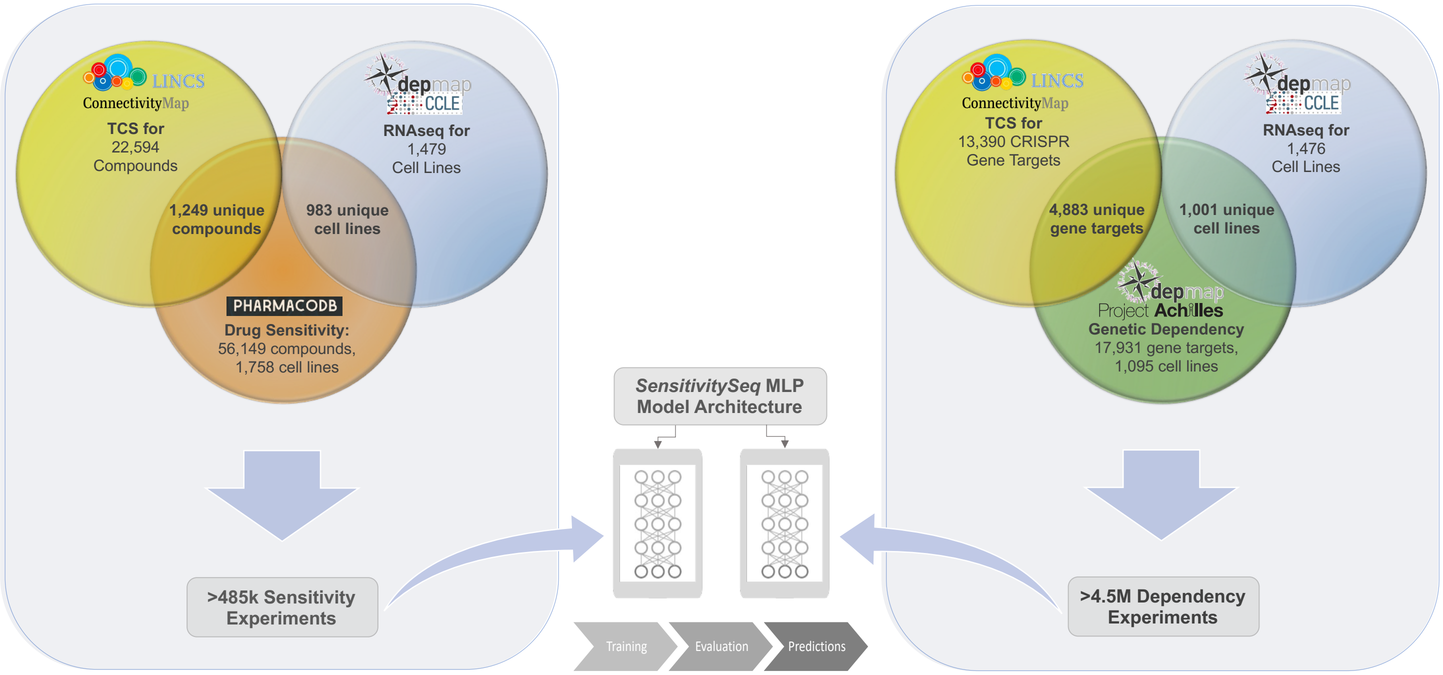

Extended Data Fig. 3. Abstract illustration summarizing aggregated datasets used for training *SensitivitySeq2.0* models. Large, aggregated datasets were used to train drug sensitivity and genetic dependency SSeq2.0 models. The *Gen2* drug sensitivity training set consisted of nearly 500,000 unique experiments across 1,249 small molecule compounds and 983 cancer cell lines, while the genetic dependency dataset spanned 4,883 CRISPR gene targets and 1,001 cancer cell lines.

Model	Loss	Accuracy	AUROC	AUPR	Precision	Recall	Specificity
**Drug Sensitivity MLP (pre-scaling)**	0.2367	89.87%	92.40%	73.08%	70.60%	58.80%	95.50%
***SSeq1.0* DS MLP**	0.2264	90.54%	93.07%	76.17%	73.46%	62.84%	95.74%
**Full CCLE Transcriptome DS MLP**	0.2410	89.89%	92.04%	74.10%	73.90%	55.74%	96.30%
**Randomized Labels**	0.4375	84.20%	48.96%	18.53%	0.00%	undefined	100.0%

Table S1. Summary of evaluation and performance for models tested during initial model development. The first model represents the performance for our initial pan-cancer Drug Sensitivity (DS) MLP model, trained and evaluated prior to scaling input datasets. The SSeq1.0 DS MLP model in row 2 reflects the performance for the primary, pan-cancer L1000-CCLE-PharmacoDB MLP model trained with scaled input data from LINCS CMap-L1000 compound TCS and CCLE gene expression signatures. The Full CCLE Transcriptome DS MLP represents the performance metrics for a model developed using the full, transcriptome-wide set of genes present in the CCLE RNAseq data as cancer cell line input features, in contrast to the 969-gene subset of the CCLE data filtered for only the L1000 landmark genes that was used for the other models. The Randomized Labels model corresponds to the SSeq1.0 MLP drug sensitivity model architecture and input features trained with ‘scrambled’ or randomly reordered outcome labels (while maintaining the imbalanced class ratio).

Model	Loss	Accuracy	AUROC	AUPR	Precision	Recall	Specificity
***SSeq1.0* DS MLP**	0.2264	90.54%	93.07%	76.17%	73.46%	62.84%	95.74%
**DS MLP, CW**	0.3412	84.91%	93.03%	75.70%	51.33%	87.64%	84.40%
**DS 1D-CNN**	0.2420	89.97%	92.01%	73.15%	72.39%	59.10%	95.77%
**DS 2D-CNN**	0.2935	89.10%	91.22%	72.84%	63.85%	71.57%	92.39%
***SSeq1.0* DS 2D-CNN, CW**	0.2691	88.84%	93.44%	76.94%	61.08%	80.93%	90.32%
**Logistic Regression**	0.4215	84.20%	66.29%	28.87%	0.00%	undefined	100.0%

Table S2. Summary of DNN Model Performance for Various Model Structures, related to Figure 6. Initial drug sensitivity (DS) model predictions were evaluated using several measures of performance as criteria. The first model listed represents the performance for our initial primary pan-cancer drug sensitivity model, SSeq1.0, as a point of comparison. An equivalent architecture to SSeq1.0 with the addition of 5:1 class weight optimization (CW) was evaluated, but this balancing strategy did not lead to better overall performance than SSeq1.0 without CW. The 2D- and 1D-CNN models represent additional early models that were trained, validated, and evaluated using the same datasets as our initial Drug Sensitivity MLP model. The 1D-CNN was not retained further due to suboptimal recall performance. The 2D-CNN architecture was evaluated with the addition of CW, leading to the retention of the CW 2D-CNN as a finalized SSeq1.0 model. In the final row, performance is shown for a basic logistic regression model trained and evaluated on the same data as prior models in the table.

Model Architecture	Evaluation	Rep	Training Allocation (%)	Testing Allocation (%)	Seed	Loss	Accuracy	AUROC	Precision	Recall	Specificity
SSeq MLP	70:30 Sample Split Set 1	1	70	30	123	0.2387	89.87%	92.31%	71.06%	58.26%	95.66%
SSeq MLP	70:30 Sample Split Set 2	2	70	30	1234	0.2367	89.87%	92.39%	72.55%	55.44%	96.17%
SSeq MLP	70:30 Sample Split Set 3	3	70	30	12345	0.2461	89.59%	92.12%	67.29%	63.47%	94.36%
SSeq MLP	70:30 Sample Split Set 4	4	70	30	123456	0.2399	89.94%	92.26%	72.10%	56.90%	95.98%
SSeq MLP	70:30 Sample Split Set 5	5	70	30	654321	0.2443	89.82%	92.15%	70.09%	60.06%	95.29%
SSeq MLP	70:30 Sample Split Sets 1-5 Mean	1-5	70	30	N/A	0.2411	89.82%	92.25%	70.62%	58.83%	95.49%
SSeq MLP	70:30 Sample Split Sets 1-5 Std Dev	1-5	70	30	N/A	0.0039	0.1352%	0.1135%	2.093%	3.104%	0.7129%

Table S3. Monte Carlo repeated random sub-sampling cross-validation with 70% of aggregated data allocated to training. Model performance results for Monte Carlo repeated random sub-sampling cross-validation models with 70% of aggregated data allocated to training are summarized.

Model Architecture	Evaluation	Rep	Training Allocation (%)	Testing Allocation (%)	Seed	Loss	Accuracy	AUROC	Precision	Recall	Specificity
SSeq MLP	1:99 Sample Split Set 1	1	1	99	123	0.3203	87.92%	86.21%	64.73%	48.36%	95.17%
SSeq MLP	1:99 Sample Split Set 2	2	1	99	1234	0.3349	87.56%	86.58%	64.35%	44.07%	95.53%
SSeq MLP	1:99 Sample Split Set 3	3	1	99	12345	0.3074	87.59%	86.54%	62.13%	50.75%	94.34%
SSeq MLP	1:99 Sample Split Set 4	4	1	99	123456	0.3034	87.30%	86.39%	61.82%	46.98%	94.69%
SSeq MLP	1:99 Sample Split Set 5	5	1	99	654321	0.3024	87.81%	86.10%	65.44%	44.85%	95.67%
SSeq MLP	1:99 Sample Split Sets 1-5 Mean	1-5	1	99	N/A	0.3137	87.64%	86.37%	63.69%	47.00%	95.08%
SSeq MLP	1:99 Sample Split Sets 1-5 Std Dev	1-5	1	99	N/A	0.01385	0.2406%	0.2086%	1.620%	2.698%	0.5620%

Table S4. Monte Carlo repeated random sub-sampling cross-validation with 1% of aggregated data allocated to training. Model performance results for Monte Carlo repeated random sub-sampling cross-validation models with 1% of aggregated data allocated to training are summarized.

Model Architecture	Evaluation	Rep	Training Allocation (%)	Testing Allocation (%)	Seed	Loss	Accuracy	AUROC	Precision	Recall	Specificity
SSeq MLP	Sample Set 1	1	10	90	123	0.2695	88.98%	90.66%	68.95%	52.32%	95.69%
SSeq MLP	Sample Set 2	2	10	90	123	0.2740	88.53%	90.40%	76.58%	37.31%	97.91%
SSeq MLP	Sample Set 3	3	10	90	123	0.2811	88.38%	90.43%	62.65%	61.68%	93.27%
SSeq MLP	Sample Set 4	4	10	90	123	0.2677	88.76%	90.67%	68.49%	50.39%	95.77%
SSeq MLP	Sample Set 5	5	10	90	123	0.2700	89.01%	90.87%	68.20%	54.49%	95.34%
SSeq MLP	Sample Set 6	6	10	90	123	0.2704	88.62%	90.87%	63.60%	61.68%	93.55%
SSeq MLP	Sample Set 7	7	10	90	123	0.2755	88.91%	90.70%	68.37%	52.91%	95.51%
SSeq MLP	Sample Set 8	8	10	90	123	0.2716	88.93%	90.71%	67.55%	54.84%	95.17%
SSeq MLP	Sample Set 9	9	10	90	123	0.2665	88.78%	90.73%	67.46%	53.22%	95.30%
SSeq MLP	Sample Set 10	10	10	90	123	0.2690	88.98%	90.81%	68.27%	53.82%	95.42%
SSeq MLP	Sample Sets 1-10 Mean	1-10	10	90	123	0.2715	88.79%	90.69%	68.01%	53.27%	95.29%
SSeq MLP	Samples Set 1-10 Std Dev	1-10	10	90	123	0.0043	0.2167%	0.1615%	3.703%	6.748%	1.268%

Table S5. 10-fold cross-validation results. Model performance results are shown for 10-fold *k-fold* cross-validation models. Ten unique slices of 10% of the total aggregated data were allocated as training sets with the remaining 90% of data used for evaluation.

CCLE Tissue Type	Sample Size	Loss	Accuracy	AUROC	AUPR	Precision	Recall	Specificity
ADRENAL CORTEX	147	0.2834	89.80%	90.09%	67.28%	63.33%	82.61%	91.13%
AUTONOMIC GANGLIA	5792	0.2862	87.62%	91.24%	73.57%	72.43%	57.08%	94.85%
BILIARY TRACT	2493	0.2245	89.93%	91.59%	62%	61.57%	48.53%	95.75%
BREAST	17150	0.2764	88.14%	89.83%	75.67%	68.29%	51.07%	95.37%
BONE	6097	0.2688	88.81%	90.95%	66.60%	74.06%	58.75%	95.45%
CENTRAL NERVOUS SYSTEM	17580	0.2212	90.52%	92.40%	70.34%	79.59%	36.97%	98.57%
CERVIX	2110	0.3032	86.30%	89.11%	66.73%	62.23%	60.42%	91.97%
ENDOMETRIUM	9087	0.2169	90.93%	93.81%	74.57%	62.70%	74.19%	93.43%
FIBROBLAST	2194	0.2069	90.70%	87.55%	43.50%	40.44%	43.79%	94.62%
HAEMATOPOIETIC AND LYMPHOID TISSUE	52836	0.3149	86.71%	90.66%	78.79%	80.20%	56.71%	95.77%
KIDNEY	7504	0.2295	90.37%	90.29%	57.29%	56.30%	58.53%	94.33%
LARGE INTESTINE	17088	0.2583	88.99%	91.13%	66.94%	60.77%	66.02%	92.84%
LIVER	7608	0.2439	89.56%	90.65%	62.46%	61.93%	57.31%	94.55%
LUNG	54175	0.2267	90.61%	92.17%	71.60%	69.99%	59.54%	95.76%
OESOPHAGUS	9935	0.2124	91.27%	93.66%	75.37%	73.83%	60.13%	96.45%
OVARY	14738	0.2135	90.98%	93.39%	72.93%	69.27%	62.84%	95.51%
PANCREAS	13444	0.1977	91.37%	93.61%	70.48%	66.92%	58.38%	95.97%
PLACENTA	312	0.3137	85.26%	85.60%	35.41%	39.53%	45.95%	90.55%
PLEURA	2875	0.2083	91.55%	92.19%	65.66%	65.37%	56.06%	96.15%
PROSTATE	1791	0.2817	88.44%	89.97%	69.41%	73.30%	52.26%	96.02%
SALIVARY GLAND	672	0.2222	89.58%	93.49%	74.75%	57.41%	72.09%	92.15%
SKIN	17169	0.2211	90.89%	91.80%	67.86%	68.80%	54.53%	96.31%
SMALL INTESTINE	183	0.4873	78.14%	88.17%	76.26%	81.82%	44.26%	95.08%
SOFT TISSUE	8701	0.2227	91.09%	93.51%	79.30%	77.65%	64.74%	96.31%
STOMACH	11224	0.2272	90.32%	92.56%	71.98%	76.11%	48.40%	97.43%
THYROID	4607	0.2198	91.19%	93.39%	76.23%	81.86%	51.76%	98.01%
UPPER AERODIGESTIVE TRACT	10916	0.2154	91.21%	93.48%	74.60%	75.84%	58.35%	96.83%
URINARY TRACT	8451	0.2025	91.91%	93.70%	74.84%	75.06%	59.75%	96.91%
Mean	10959.96	0.2502	89.36%	91.43%	68.66%	67.74%	57.18%	95.15%
Std Dev	13275.33	0.0583	2.778%	2.099%	9.836%	10.72%	9.615%	1.975%

Table S6. Cell line cross-domain validation model performance by CCLE Tissue Type, related to Figure 4.

Ligand Type	Loss	Accuracy	AUROC	Precision	Recall	Specificity	Unique Compounds	Test Set Size
Activator	0.3135	89.34%	93.29%	84.06%	93.43%	86.14%	7	8939
Agonist	0.1561	95.74%	87.79%	80.88%	56.47%	98.92%	18	13108
Antagonist	0.2523	88.84%	42.36%	1.63%	3.04%	92.41%	17	10738
Blocker[a]	0.0126	100%	100.0%	100.0%	100.0%	100.0%	1	36
Enhancer	0.1284	96.77%	93.93%	81.58%	83.78%	98.08%	3	804
Inhibitor	0.5633	81.50%	75.73%	48.77%	59.95%	86.22%	355	325799
Ligand—other/unknown	0.3006	90.50%	71.27%	0%	undefined	100.0%	1	926
Modulator	0.1004	98.82%	35.00%	0%	undefined	100.0%	1	929
Stimulant	0.4078	79.98%	87.00%	69.21%	99.37%	64.34%	3	2123
Stabilizing Agent	0.7027	57.56%	42.84%	58.46%	97.39%	0.00%	2	714
Mean	0.2938	87.91%	72.92%	52.46%	74.18%	82.61%	40.80	36411.60
Std Dev	0.2155	12.65%	24.26%	38.49%	33.55%	31.08%	110.59	101795.74

[a]no positive classes present in test set

Table S7. Cross-domain validation by compound ligand category.

Model	Loss	Accuracy	AUROC	AUPR	Precision	Recall	Specificity
**Balanced by Exclusion (*SSeq1.0* MLP)**	0.3734	84.14%	92.59%	73.94%	49.91%	87.71%	83.48%
**Balanced by Fusion (*SSeq1.0* MLP)**	0.3313	85.88%	93.34%	76.42%	53.24%	87.37%	85.59%
***SSeq1.0* DS MLP, CW**	0.3412	84.91%	93.03%	75.70%	51.33%	87.64%	84.40%

Table S8. Model performance after applying various balancing strategies, related to Figure 6. Model performance results are shown following application of three balancing strategies tested. An exclusion-based balancing strategy (row 1), in which training set experiments with negative (resistant) outcomes were randomly excluded until reaching approximately equal, 1:1 proportions of experiments with resistant outcomes to those with sensitive outcomes. In row 2, a fusion-based balancing strategy was applied by randomly splitting the resistant-outcome training set experiments into five groups, with each subset approximately proportional to the sensitive-outcome training set experiments subset. Subsequently, each resistant-outcome subset was separately combined with the sensitive-outcome subset to form five smaller, balanced training data subsets. Training subsets were used consecutively to train a fusion MLP model in a series of five training steps, for 5 epochs per subset to equal 25 total training epochs. In row 3, a class-weights, hyperparameter-based strategy was used to train each model with 5:1 class weights set for sensitive:resistant classes. Setting class weights to 5:1 leads to each sensitive-outcome experiment exerting the same level of influence on model weights as five resistant-outcome experiments during training. All models were evaluated using the same separate, unaltered, and untransformed test set.

Model	Loss	Accuracy	AUROC	AUPR	Precision	Recall	Specificity
***SSeq1.0* CCLE-only Input MLP**	0.4304	84.20%	60.00%	22.18%	0.00%	undefined	100.0%
***SSeq1.0* L1000-only Input MLP**	0.2540	89.32%	91.38%	70.21%	71.24%	54.35%	95.88%
**CCLE Protein Quantification MLP**	0.2229	90.65%	93.09%	74.50%	70.61%	65.40%	95.15%
***SSeq2.0* Genetic Dependency MLP**	0.1670	92.86%	98.44%	90.49%	55.28%	95.44%	92.61%

Table S9. Comparison of DNN Models for Various Input Features and Datasets, related to Figure 6.

Model	Loss	Accuracy	AUROC	AUPR	Precision	Recall	Specificity
**TCS, TPM Dual-Subnetwork MLP**	0.2532	89.49%	91.36%	73.10%	75.55%	53.38%	96.60%
**ECFP4, TCS, TPM 3-Subnetwork MLP**	0.2330	90.10%	93.25%	76.74%	78.46%	54.95%	97.03%
**ECFP4, TPM Dual-Subnetwork MLP**	0.2379	89.57%	93.13%	76.16%	82.20%	46.73%	98.01%

Table S10. Comparison of performance for drug sensitivity models trained on an ECFP4-annotated subset of the initial aggregated dataset.

Model	Accuracy	AUROC	AUPR	Precision	Recall	Specificity	True Positives	True Negatives	False Positives	False Negatives
**PC Validation, *SSeq1.0* MLP**	59.87%	70.72%	88.30%	83.70%	57.42%	67.09%	267	106	52	198
**PC Validation, *SSeq2.0* MLP**	67.42%	65.87%	85.83%	74.17%	86.45%	11.39%	402	18	140	63

Table S11. Prospective, external PC validation of *SensitivitySeq*. SSeq drug sensitivity MLP models were validated in prostate cancer (PC) cell lines. SSeq2.0 was validated using a prospective, single-tissue experimental validation. SSeq1.0 was also externally validated using the same dataset for comparison. RNAseq TPM for eight cell lines with three biological replicates each were used as input to generate predictions for each cell line for each L1000 TCS compound available. Following *in vitro* validation experiments, a 5.0 μM IC50 cutoff was applied to determine actual classes and evaluate predicted classes.
