# Supplementary material for: Pan-Cancer Drug Sensitivity Prediction from Gene Expression using Deep Learning
Source: bioRxiv. 2024 Nov 15:2024.11.15.623715. Preprint. [Version 1] doi: 10.1101/2024.11.15.623715 (PMC11601385; doi:10.1101/2024.11.15.623715)
Supplement: Supplement 2 — Table S12. External Prostate Cancer Predictions/Validation Table, related to Table S11. [file media-2.pdf]

Cell line	Compound	IC50 (actual)	Actual score	SSeq1.0 prediction estimate	SSeq1.0 predicted score	SSeq2.0 prediction estimate	SSeq2.0 predicted score
22Rv1	afatinib	1.88	1	0.142782360	0	0.538164198	1
C4-2B	afatinib	5.45	0	0.149451077	0	0.690935493	1
DU145	afatinib	2.25	1	0.149314344	0	0.542400181	1
LNCaP	afatinib	1.22	1	0.071935117	0	0.689906955	1
NCI-H660	afatinib	7.82	0	0.021622747	0	0.538164198	1
PC3	afatinib	3.95	1	0.206208199	0	0.561330438	1
RWPE-1	afatinib	0.09	1	0.384666979	0	0.538164198	1
VCaP	afatinib	23.6	0	0.049034894	0	0.539431930	1
22Rv1	alisertib	30	0	0.412455201	0	0.558874667	1
C4-2B	alisertib	30	0	0.562531710	1	0.711332798	1
DU145	alisertib	13.45	0	0.217999965	0	0.558874667	1
LNCaP	alisertib	20.59	0	0.355319440	0	0.705398202	1
NCI-H660	alisertib	30	0	0.586519897	1	0.558874667	1
PC3	alisertib	16.94	0	0.107419848	0	0.560064554	1
RWPE-1	alisertib	10.06	0	0.268422067	0	0.558874667	1
VCaP	alisertib	30	0	0.297763586	0	0.560448468	1
22Rv1	alvocidib	0.06	1	0.917779446	1	0.897899508	1
C4-2B	alvocidib	0	1	0.936987877	1	0.957896471	1
DU145	alvocidib	0	1	0.878314674	1	0.901111364	1
LNCaP	alvocidib	0	1	0.894602180	1	0.957792997	1
NCI-H660	alvocidib	30	0	0.822813869	1	0.897899508	1
PC3	alvocidib	0	1	0.807090402	1	0.915017545	1
RWPE-1	alvocidib	0	1	0.908599854	1	0.897899508	1
VCaP	alvocidib	0	1	0.826730430	1	0.900076807	1
22Rv1	anisomycin	0	1	0.535566986	1	0.780103922	1
C4-2B	anisomycin	0	1	0.609309793	1	0.899583340	1
DU145	anisomycin	0.01	1	0.393346429	0	0.783417702	1
LNCaP	anisomycin	0.01	1	0.485879332	0	0.899318457	1
NCI-H660	anisomycin	30	0	0.427598000	0	0.780103922	1
PC3	anisomycin	0.08	1	0.283856273	0	0.805271149	1
RWPE-1	anisomycin	0.01	1	0.433127880	0	0.780103922	1
VCaP	anisomycin	5.12	0	0.429514557	0	0.780978084	1
22Rv1	BI-2536	0	1	0.943608165	1	0.874229670	1
C4-2B	BI-2536	0	1	0.958806992	1	0.947653890	1
DU145	BI-2536	0	1	0.910637140	1	0.878232718	1
LNCaP	BI-2536	0	1	0.925457656	1	0.947536826	1
NCI-H660	BI-2536	4.45	1	0.883519888	1	0.874229670	1
PC3	BI-2536	0	1	0.811250925	1	0.894525409	1
RWPE-1	BI-2536	0	1	0.935109138	1	0.874229670	1
VCaP	BI-2536	0	1	0.876823068	1	0.876939058	1
22Rv1	cabazitaxel	30	0	0.725094736	1	0.955084205	1
C4-2B	cabazitaxel	0	1	0.769216478	1	0.982285380	1
DU145	cabazitaxel	0	1	0.679298043	1	0.956856966	1
LNCaP	cabazitaxel	0	1	0.684690356	1	0.982240796	1
NCI-H660	cabazitaxel	30	0	0.514086246	1	0.955084205	1
PC3	cabazitaxel	0	1	0.614686549	1	0.963878632	1
RWPE-1	cabazitaxel	0	1	0.710921526	1	0.955084205	1
VCaP	cabazitaxel	0	1	0.597662449	1	0.956288099	1
22Rv1	cladribine	0.31	1	0.518917680	1	0.752118945	1
C4-2B	cladribine	0.12	1	0.578958452	1	0.878757596	1
DU145	cladribine	1.85	1	0.367065161	0	0.755814433	1
LNCaP	cladribine	0.14	1	0.410308838	0	0.878445268	1
NCI-H660	cladribine	180	0	0.276999652	0	0.752118945	1
PC3	cladribine	22.39	0	0.266851485	0	0.752966881	1
RWPE-1	cladribine	0.28	1	0.438352138	0	0.752118945	1
VCaP	cladribine	30	0	0.345904648	0	0.753245115	1
22Rv1	danusertib	30	0	0.07794854	0	0.767408252	1
C4-2B	danusertib	0	1	0.091372281	0	0.890387833	1
DU145	danusertib	1.22	1	0.048509479	0	0.770642757	1
LNCaP	danusertib	0	1	0.071571082	0	0.890101612	1
NCI-H660	danusertib	30	0	0.087356687	0	0.767408252	1
PC3	danusertib	45.37	0	0.029868245	0	0.768267870	1
RWPE-1	danusertib	0.83	1	0.053665698	0	0.767408252	1
VCaP	danusertib	30	0	0.077460647	0	0.768509865	1
22Rv1	dasatinib	24.63	0	0.672664464	1	0.587255418	1
C4-2B	dasatinib	11.85	0	0.680742025	1	0.742329836	1
DU145	dasatinib	0.21	1	0.683224559	1	0.591964960	1
LNCaP	dasatinib	14.86	0	0.583333731	1	0.738205552	1
NCI-H660	dasatinib	30	0	0.148270190	0	0.587255418	1
PC3	dasatinib	22.74	0	0.652873993	1	0.588304222	1
RWPE-1	dasatinib	0.48	1	0.708958447	1	0.587255418	1
VCaP	dasatinib	30	0	0.293738723	0	0.588645995	1
22Rv1	delanzomib	3.31	1	0.515109599	1	0.944406748	1
C4-2B	delanzomib	0	1	0.597142160	1	0.978011966	1
DU145	delanzomib	2.98	1	0.368071824	0	0.946456015	1
LNCaP	delanzomib	0.25	1	0.461424947	0	0.977956831	1
NCI-H660	delanzomib	0.04	1	0.396050096	0	0.944406748	1
PC3	delanzomib	5.48	0	0.260596097	0	0.944864273	1
RWPE-1	delanzomib	0	1	0.425655454	0	0.944406748	1
VCaP	delanzomib	0	1	0.403796315	0	0.945749938	1
22Rv1	docetaxel	0	1	0.743937314	1	0.805332005	1
C4-2B	docetaxel	0	1	0.759613454	1	0.915802717	1
DU145	docetaxel	0	1	0.734765053	1	0.810435236	1
LNCaP	docetaxel	0	1	0.704077244	1	0.915620685	1
NCI-H660	docetaxel	30	0	0.272650301	0	0.805332005	1
PC3	docetaxel	0	1	0.699551582	1	0.832469940	1
RWPE-1	docetaxel	0	1	0.754433155	1	0.805332005	1
VCaP	docetaxel	0	1	0.562275946	1	0.808715940	1
22Rv1	doxorubicin	1.76	1	0.917484283	1	0.933674097	1
C4-2B	doxorubicin	2.32	1	0.937280893	1	0.973536968	1
DU145	doxorubicin	2.67	1	0.873442113	1	0.935979366	1
LNCaP	doxorubicin	0.7	1	0.891810477	1	0.973470867	1
NCI-H660	doxorubicin	3.52	1	0.825995564	1	0.933674097	1
PC3	doxorubicin	2.61	1	0.796601772	1	0.945927560	1
RWPE-1	doxorubicin	0.25	1	0.905400634	1	0.933674097	1
VCaP	doxorubicin	1.83	1	0.822135806	1	0.935229301	1
22Rv1	foretinib	0	1	0.520031989	1	0.626131713	1
C4-2B	foretinib	3.94	1	0.614345908	1	0.776917577	1
DU145	foretinib	1.15	1	0.352992982	0	0.630936027	1
LNCaP	foretinib	0	1	0.458470374	0	0.776169538	1
NCI-H660	foretinib	12	0	0.467473716	0	0.626131713	1
PC3	foretinib	0.94	1	0.245098054	0	0.649018824	1
RWPE-1	foretinib	1.8	1	0.408249021	0	0.626131713	1
VCaP	foretinib	0.168	1	0.400226921	0	0.629600048	1
22Rv1	irinotecan	1.09	1	0.521790862	1	0.499655545	0
C4-2B	irinotecan	7.7	0	0.601659656	1	0.657574058	1
DU145	irinotecan	2.09	1	0.377734035	0	0.506911993	1
LNCaP	irinotecan	2.08	1	0.466879964	0	0.655854404	1
NCI-H660	irinotecan	30	0	0.404456347	0	0.499655545	0
PC3	irinotecan	73.7	0	0.269218087	0	0.499655545	0
RWPE-1	irinotecan	2.94	1	0.431501299	0	0.499655545	0
VCaP	irinotecan	0.21	1	0.405478597	0	0.504889667	1
22Rv1	lestaurtinib	1.02	1	0.631889641	1	0.720049024	1
C4-2B	lestaurtinib	0	1	0.664161921	1	0.857274532	1
DU145	lestaurtinib	0.66	1	0.616400003	1	0.725078881	1
LNCaP	lestaurtinib	0.16	1	0.607629836	1	0.856915832	1
NCI-H660	lestaurtinib	16.95	0	0.323508173	0	0.720049024	1
PC3	lestaurtinib	0.99	1	0.486676842	0	0.741457582	1
RWPE-1	lestaurtinib	0.34	1	0.621448755	1	0.720049024	1
VCaP	lestaurtinib	30	0	0.479613513	0	0.721600533	1
22Rv1	mitoxantrone	0.15	1	0.999408245	1	0.775885582	1
C4-2B	mitoxantrone	0.3	1	0.999579608	1	0.896502733	1
DU145	mitoxantrone	0	1	0.998866916	1	0.778867543	1
LNCaP	mitoxantrone	0.08	1	0.999170661	1	0.896283746	1
NCI-H660	mitoxantrone	1.77	1	0.998643041	1	0.775885582	1
PC3	mitoxantrone	0.68	1	0.997340024	1	0.799872756	1
RWPE-1	mitoxantrone	0.22	1	0.999204516	1	0.775885582	1
VCaP	mitoxantrone	0.66	1	0.998522580	1	0.777858913	1
22Rv1	MK-1775	1.53	1	0.309861064	0	0.564128220	1
C4-2B	MK-1775	0.62	1	0.371170461	0	0.717569947	1
DU145	MK-1775	0.93	1	0.216849953	0	0.567659974	1
LNCaP	MK-1775	0.66	1	0.256115049	0	0.716593683	1
NCI-H660	MK-1775	1335.2	0	0.305694252	0	0.564128220	1
PC3	MK-1775	1.11	1	0.143258214	0	0.588397324	1
RWPE-1	MK-1775	20.97	0	0.251462638	0	0.564128220	1
VCaP	MK-1775	30	0	0.252946675	0	0.565177321	1
22Rv1	MLN-0128	0	1	0.680450320	1	0.469782561	0
C4-2B	MLN-0128	0	1	0.715497196	1	0.639645100	1
DU145	MLN-0128	0	1	0.654029965	1	0.469782561	0
LNCaP	MLN-0128	0	1	0.651003242	1	0.637790620	1
PC3	MLN-0128	0	1	0.572612405	1	0.515787244	1
RWPE-1	MLN-0128	0	1	0.668436527	1	0.469782561	0
VCaP	MLN-0128	30	0	0.581978798	1	0.476449728	0
22Rv1	panobinostat	0.04	1	0.997586012	1	0.971533060	1
C4-2B	panobinostat	0.03	1	0.998270631	1	0.988816619	1
DU145	panobinostat	0	1	0.995521784	1	0.972703636	1
LNCaP	panobinostat	0	1	0.996653855	1	0.988788247	1
NCI-H660	panobinostat	0	1	0.994546354	1	0.971533060	1
PC3	panobinostat	0.01	1	0.989740372	1	0.977001488	1
RWPE-1	panobinostat	0.17	1	0.996853232	1	0.971533060	1
VCaP	panobinostat	30	0	0.994156480	1	0.971885502	1
22Rv1	PD-0325901	30	0	0.528346896	1	0.485723734	0
C4-2B	PD-0325901	30	0	0.557128012	1	0.648250937	1
DU145	PD-0325901	5.22	0	0.554656148	1	0.500000000	1
LNCaP	PD-0325901	30	0	0.344728231	0	0.641564846	1
NCI-H660	PD-0325901	19.7	0	0.074462116	0	0.485723734	0
PC3	PD-0325901	30	0	0.610787153	1	0.487786978	0
RWPE-1	PD-0325901	0	1	0.677488804	1	0.485723734	0
VCaP	PD-0325901	30	0	0.167334914	0	0.492406249	0
22Rv1	rotenone	0	1	0.530090094	1	0.611292601	1
C4-2B	rotenone	0	1	0.621801853	1	0.763253093	1
DU145	rotenone	0.244	1	0.361981481	0	0.616725922	1
LNCaP	rotenone	0	1	0.476940334	0	0.762495756	1
NCI-H660	rotenone	40.4	0	0.486687064	0	0.611292601	1
PC3	rotenone	0	1	0.251586825	0	0.635373592	1
RWPE-1	rotenone	0.0943	1	0.418896914	0	0.611292601	1
VCaP	rotenone	26.3	0	0.415680200	0	0.612936735	1
22Rv1	TAK-733	30	0	0.508037686	1	0.618616700	1
C4-2B	TAK-733	30	0	0.561559737	1	0.770852149	1
DU145	TAK-733	11.9	0	0.324154258	0	0.618616700	1
LNCaP	TAK-733	30	0	0.288563192	0	0.766088843	1
NCI-H660	TAK-733	30	0	0.138903528	0	0.618616700	1
PC3	TAK-733	27.08	0	0.259862334	0	0.619811952	1
RWPE-1	TAK-733	0	1	0.437957048	0	0.618616700	1
VCaP	TAK-733	30	0	0.219015837	0	0.620191157	1
22Rv1	taselisib	2.05	1	0.608039081	1	0.266572446	0
C4-2B	taselisib	0.93	1	0.624543667	1	0.527704298	1
DU145	taselisib	3.81	1	0.589558959	1	0.266572446	0
LNCaP	taselisib	0.17	1	0.508957148	1	0.525076687	1
NCI-H660	taselisib	0.62	1	0.186033905	0	0.266572446	0
PC3	taselisib	1.67	1	0.410802335	0	0.267707437	0
RWPE-1	taselisib	0.32	1	0.604354382	1	0.266572446	0
VCaP	taselisib	1.02	1	0.313981295	0	0.268074363	0
22Rv1	teniposide	0.741	1	0.650498807	1	0.714690924	1
C4-2B	teniposide	2.97	1	0.760117710	1	0.853410304	1
DU145	teniposide	0	1	0.401435852	0	0.719863176	1
LNCaP	teniposide	1.04	1	0.585485756	1	0.853043497	1
NCI-H660	teniposide	0.29	1	0.678818345	1	0.714690924	1
PC3	teniposide	0.601	1	0.215558469	0	0.736995697	1
RWPE-1	teniposide	0.118	1	0.448394448	0	0.714690924	1
VCaP	teniposide	4.8	1	0.506501496	1	0.718193054	1
22Rv1	tivantinib	0.3	1	0.369169384	0	0.668236256	1
C4-2B	tivantinib	0.77	1	0.490735978	0	0.814820290	1
DU145	tivantinib	0.45	1	0.179183096	0	0.671793818	1
LNCaP	tivantinib	0.34	1	0.302020311	0	0.814270616	1
NCI-H660	tivantinib	30	0	0.530208409	1	0.668236256	1
PC3	tivantinib	30	0	0.072216481	0	0.669039249	1
RWPE-1	tivantinib	0.09	1	0.218029916	0	0.668236256	1
VCaP	tivantinib	0.31	1	0.256141901	0	0.670608759	1
22Rv1	vorinostat	1.66	1	0.249474913	0	0.364392698	0
C4-2B	vorinostat	0.85	1	0.305279911	0	0.583018184	1
DU145	vorinostat	0.88	1	0.161108434	0	0.364392698	0
LNCaP	vorinostat	0.84	1	0.211882234	0	0.581072569	1
NCI-H660	vorinostat	1.62	1	0.297014743	0	0.364392698	0
PC3	vorinostat	4.86	1	0.103281438	0	0.365933567	0
RWPE-1	vorinostat	1.32	1	0.181291431	0	0.364392698	0
VCaP	vorinostat	2.69	1	0.207778007	0	0.366428792	0
